# Supplementary material for: Prognostic Significance of the Myelodysplastic Syndrome-Specific Comorbidity Index (MDS-CI) in Patients with Myelofibrosis: A Retrospective Study
Source: Cancers (Basel). 2023 Sep 24;15(19):4698. doi: 10.3390/cancers15194698 (PMC10571648; doi:10.3390/cancers15194698)
Supplement: Supplementary file 1 [file cancers-15-04698-s001.zip › cancers-2586331-supplementary.pdf]

Supplementary materials

Supplementary methods

DNA was isolated from bone marrow aspirate or biopsy samples by automated DNA purification on a Maxwell® RSC instrument (Promega, Fitchburg, WI, USA). DNA concentration was measured with the Qubit™ dsDNA BR (Broad Range) Assay Kit (Thermo Fisher Scientific, San Francisco, CA, USA). For NGS-based mutation analysis, the DNA panel of the Oncomine™ Myeloid Research Assay (OMRA; Thermo Fisher Scientific,) was used that targets all exonic regions of 17 genes and exonic hotspots of 23 genes (526 amplicons). OMRA libraries were prepared manually according to the manufacturer's instructions (Thermo Fisher Scientific). Briefly, barcoded libraries were generated from 30 ng of sample genomic DNA. Targets were amplified using multiplex PCR. Amplification products were partially digested with Fupa enzyme, followed by ligation of unique barcode adapters for each library. The barcoded libraries were quantified by qPCR using the KAPA Library Quantification Kit (Roche) and diluted to 40 pmol/L. Templating and clonal amplification of sample libraries onto Ion Sphere Particles by emulsion PCR were performed with the Ion Chef™ instrument (Thermo Fisher Scientific). Enriched Ion Sphere Particles were loaded onto 510, 520, or 530 chips using an Ion 510™ & Ion 520™, or Ion 530™ Kit. A total of 3, 4 and 20 samples per 510, 520 and 530 chip, respectively, were run in the automated templating preparation on the Ion Chef™. Sequencing was performed on an Ion GeneStudio™ S5 instrument (Thermo Fisher Scientific). The Torrent Suite software version 5.10.0 (Thermo Fisher Scientific) was used for sequence alignment to reference genome hg19 and base calling. Coverage maps were generated using the Coverage Analysis plugin version 5.16.0 (Thermo Fisher Scientific). Variant identification and annotation were performed using Ion Reporter™ (IR) software version 5.16 (Thermo Fisher Scientific) using the default analysis parameter settings of the Oncomine Myeloid Research workflow. In these settings, the minimum coverage requirement for the analysis is 20 for both SNVs and indels, and 15 for hotspots; the minimum cutoff variant allele fraction (VAF) is 2.5% for both SNVs and indels and 3% for hotspots; and the maximum strand bias tolerance is 0.9 for SNVs, 0.85 for indels, and 0.96 for hotspots.

Supplementary Table S1. Variables and risk groups of the MDS-CI according to [17].

**Comorbidity**	**Definition**	**Points**
Cardiac	Arrhythmia (atrial fibrillation or flutter, sick sinus syndrome or ventricular arrhythmias)<br>Heart valve disease (except mitral valve prolapse)<br>Coronary artery disease or myocardial infarction (one or more vessel-coronary artery stenosis requiring medical treatment, stent or bypass graft)<br>Congestive heart failure or ejection fraction ≤ 50%	2
Moderate to severe hepatic disease	Cirrhosis<br>Fibrosis<br>Persistent bilirubin >1.5x ULN<br>AST/ALT >1.5x ULN	1
Severe pulmonary disease	DLCO and/or FEV1 ≤ 65% or<br>Dyspnoe at rest<br>or requires oxygen	1
Renal disease	Persistent creatinine >2mg/dl<br>renal dialysis or renal transplant	1
Solid tumor	Malignancy at any time point in the patients history, excluding non-melanoma skin cancer	1
**MDS-Comorbidity Index**		
MDS-CI risk	Sum of individual variable scores	
Low risk	0	
Intermediate risk	1-2	
High risk	>2	

DLCO indicates diffusion capacity of the lung for carbon monoxide; FEV1: forced expiratory volume in one second; ULN upper limit of normal; AST: aspartate aminotransferase; ALAT: Alanine aminotransferase

Supplementary Table S2. Characteristics of patients within the MDS-CI high-risk group (n = 7).

UPN	Age at diagnosis	DIPSS	Year of diagnosis	Status	Treatment	Cause of Death	Comment
77	80	high	2020	deceased	Ruxolitinib	Sepsis in Neutropenia	
33	78	high	2012	deceased	Hydroxyurea followed by Ruxolitinib	Pneumonia with multi organ failure	
21	73	Int-2	2020	deceased	Best Supportive Care including transfusions	Multi organ failure during chemotherapy with carboplatin/etoposide for a metastatic NET, diagnosed simultaneously with post-PV MF.	Severe thrombocytopenia prevented use of Ruxolitinib
72	76	Int-2	2010	deceased	Best Supportive Care including transfusions	Decompensated liver cirrhosis	Ruxolitinib not yet available
28	76	Int-2	2019	alive	Hydroxurea followed by Ruxolitinib	Not applicable	
68	69	Int-1	2021	alive	Ruxolitinib	Not applicable	
35	74	Int-1	2017	alive	Ruxolitinib	Not applicable	

Supplementary Table S3.

Characteristics of patients with primary myelofibrosis. Data are shown for the whole population and according to the presence or absence of comorbidities as defined by the MDS-CI (MDS-CI 0 versus MDS-CI ≥ 1).

	**Primary Myelofibroses**	**MDS-CI 0**	**MDS-CI ≥1**	p
n	51	26	25	
Age [years], median, (IQR)	73 (62; 78)	71 (59;77)	76 (64; 80)	0.124
Female n, (%)	23 (45)	12 (46)	11 (44)	0.877
Bone Marrow fibrosis grade 2 n (%)	37 (72.5)	16 (61.5)	21 (84)	
Bone Marrow fibrosis grade 3 (n, %)	14 (27.5)	10 (38.5)	4 (16)	0.072
Hemoglobin [g/l], median (IQR)	111 (93; 124)	114 (99; 124)	107 (86; 126)	0.386
Platelet count (× 10^9/l), median (IQR)	515 (218; 762)	551 (197; 791)	434 (263; 717)	0.749
Leukocytes (× 10^9/l), median (IQR)	9.4 (6.8; 14.4)	7..45 (6.65; 11.48)	11.9 (7.5; 21)	**0.041**
Neutrophils (× 10^9/l), median (IQR)	6.5 (4.3; 12.7)	5.8 (3.88; 7.62)	9.8 (4.85; 16.45)	0.085
Monocytes (× 10^9/l), median (IQR)	0.56 (0.36; 0.85)	0.55 (0.40; 0.76)	0.57 (0.31; 1.18)	0.816
Lymphocytes (× 10^9/l), median (IQR)	1.60 (1.1; 2.20)	1.55 (1.15; 1.93)	1.70 (1.05; 2.30)	0.391
Blasts PB (%), median IQR	0 (0;1)	0 (0;0.25)	0 (0, 1)	0.090
Constitutional Symptoms (n, %)	26 (51)	11 (42.3)	15 (60)	0.206
LDH available [U/l] median (IQR)	47/51 502 (362;645)	24/26 490 (364; 5932)	23/25 554 (326; 777)	0.469
CRP available [mg/l], median (IQR)	49/51 5 (2;16)	24/26 3.0 (1.0; 6.5)	25/25 9.0 (4.5; 33.5)	**0.002**
Ferritin available [µg/l], Median (IQR)	35/51 177 (107; 300)	17/26 130 (59; 175)	18/25 277 (170; 458)	**0.002**
Albumin available [g/l], median (IQR)	43/51 40.2 (37; 42.3)	23/26 42.1 (37.8; 43.6)	20/25 38.1 (36.4; 42.1)	0.062
Need of transfusion (n, %)	15 (29.4)	7 (26.9)	8 (32)	0.691
Splenomegaly (clinically or imaging)	42 (82.4)	21 (80.8)	21 (84)	0.762
BMI available [kg/m^2], median (IQR)	47/51 24.4 (21.5; 28.2)	22/26 23.0 (21.4; 28.1)	25/25 26.0 (21.6; 28.6)	0.430

Supplementary Table S4.

Multivariate Cox regression models, including the MDS-CI and “DIPSSdich” (Multivariate 1, n=51) or “MIPSS70dich” (Multivariate 2, n=41) as categorical variables for patients with primary myelofibrosis only.

	**Multivariate 1**			**Multivariate 2**		
	HR	95% CI	p	HR	95% CI	p
**MDS-CI***						
intermediate	1.80	0.65; 4.96	0.2562	1.81	0.829; 3.93	0.1370
high	15.41	42.246; 105.8	0.0054	11.9	2.42; 58.76	0.0023
**DIPSSdich****	7.39	200; 27.32	0.0027			
**MIPSS70dich*****				4.79	1.56; 14.75	0.0063

*Reference low risk; **Reference low/intermediate-1 risk; ***Reference low/intermediate risk

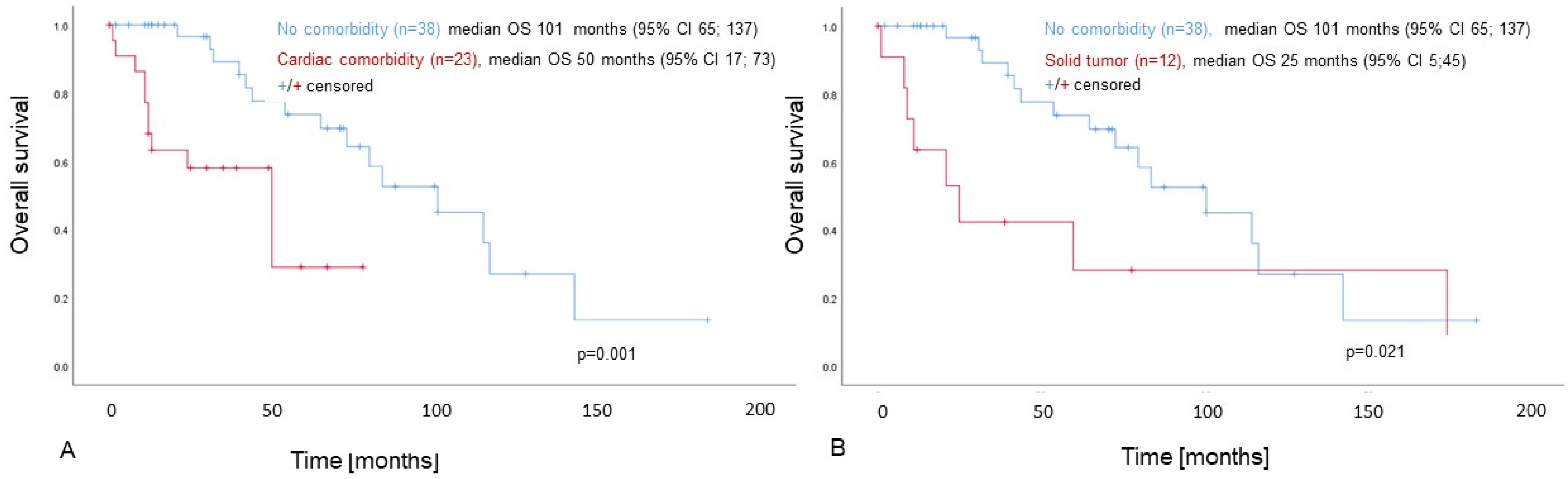

Supplementary Figure S1. Survival of MF patients with a cardiac comorbidity (A) or a solid tumor (B) compared to MF patients without any comorbidities according to MDS-CI.

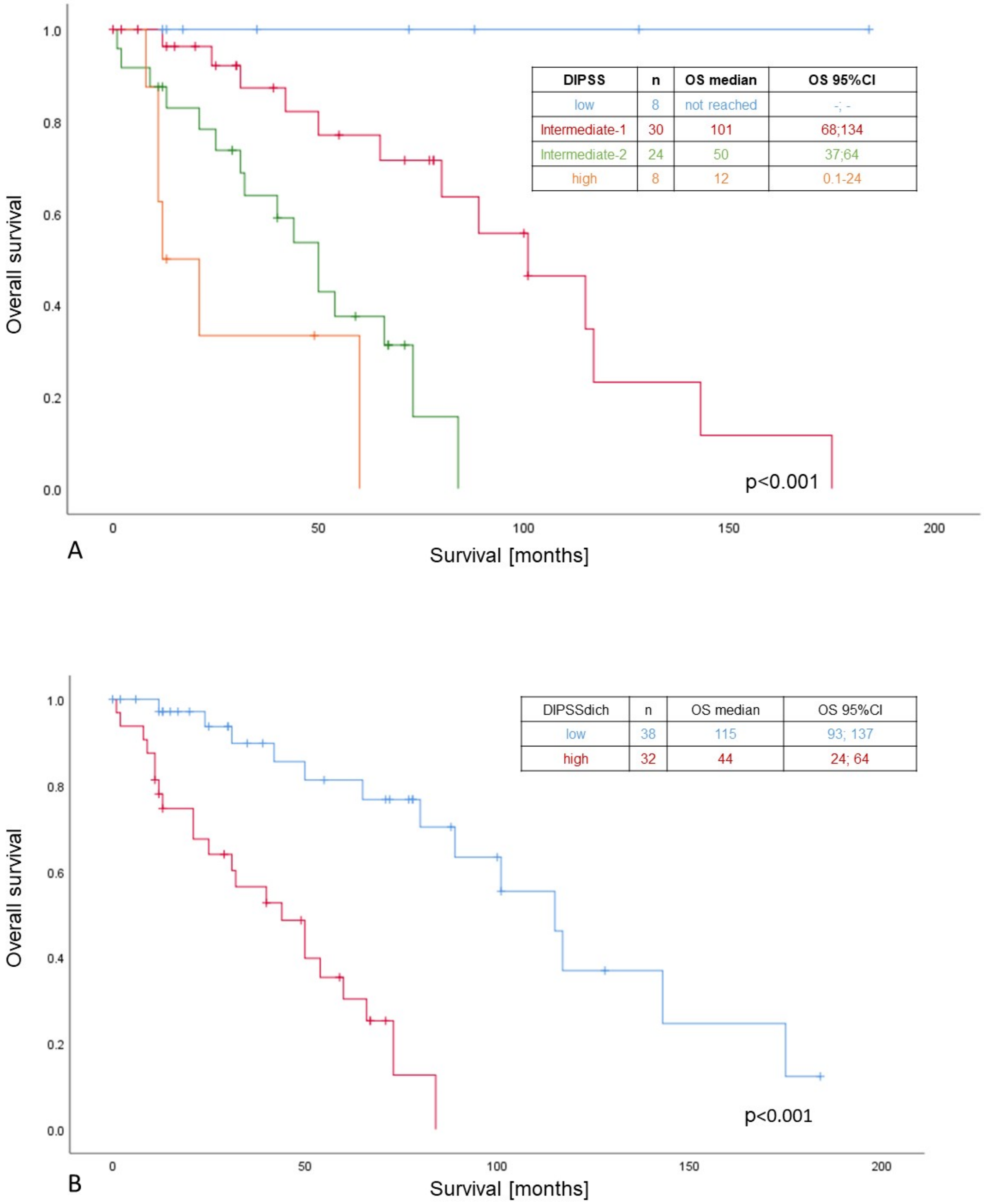

Supplementary Figure S2. Survival according to DIPSS (A) and DIPSSdich (B), with DIPSSdich low comprising low and intermediate-1 and DIPSSdich high comprising intermediate-2 and high risk patients according to DIPSS.

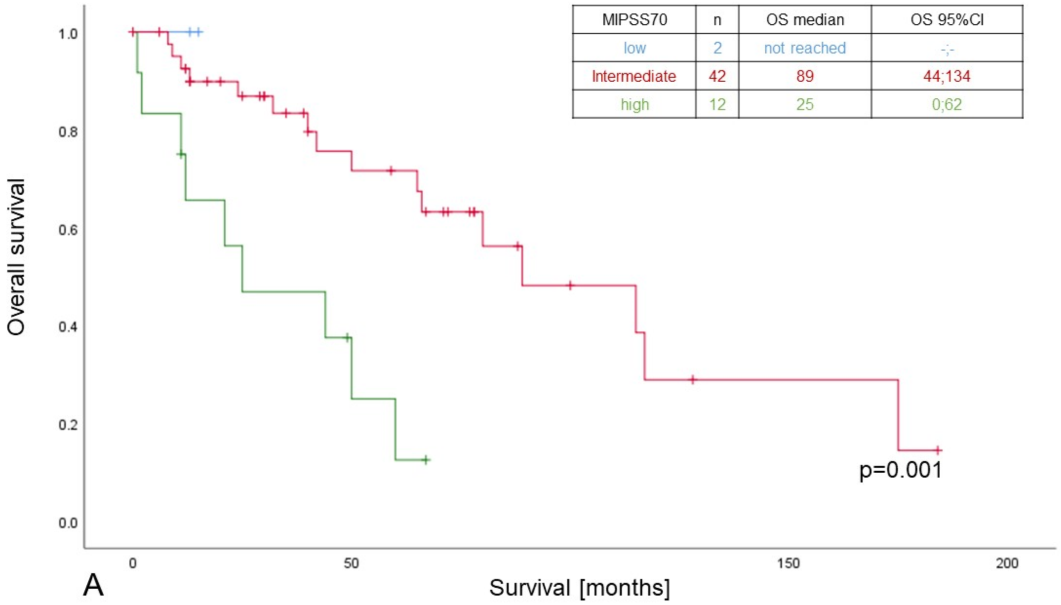

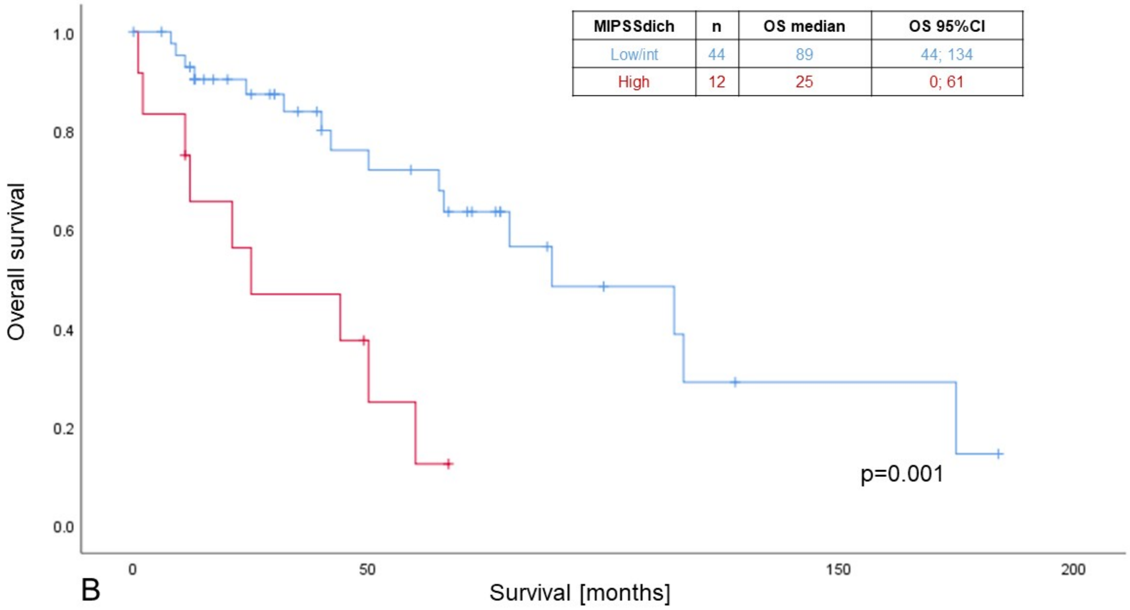

Supplementary Figure S3. Survival of the MF cohort according to MIPSS70 (A) and MIPSS70dich (B), with $MIPSS70dich^{low/int}$ comprising low and intermediate-risk patients and $MIPSS70dich^{high}$ comprising high-risk patients according to MIPSS70.

Reference

17 Della Porta, M.G.; Malcovati, L.; Strupp, C.; Ambaglio, I.; Kuendgen, A.; Zipperer, E.; Travaglino, E.; Invernizzi, R.; Pascutto, C.; Lazzarino, M.; et al. Risk Stratification Based on Both Disease Status and Extra-Hematologic Comorbidities in Patients with Myelodysplastic Syndrome. *Haematologica* **2011**, *96*, 441–449. https://doi.org/10.3324/haematol.2010.033506.
